# Cervical cancer isolate PT3, super-permissive for adeno-associated virus replication, over-expresses DNA polymerase δ, PCNA, RFC and RPA

**DOI:** 10.1186/1471-2180-9-79

**Published:** 2009-04-23

**Authors:** Bum Yong Kang, Hong You, Sarmistha Bandyopadhyay, Nalini Agrawal, Russell B Melchert, Alexei G Basnakian, Yong Liu, Paul L Hermonat

**Affiliations:** 1 Department of Internal Medicine, Gene Therapy Program, University of Arkansas for Medical Sciences, 4301 West Markham St, Little Rock, AR 72205, USA; 2 Obstetrics and Gynecology, University of Arkansas for Medical Sciences, 4301 West Markham St, Little Rock, AR 72205, USA; 3 Pharmaceutical Sciences, University of Arkansas for Medical Sciences, 4301 West Markham St, Little Rock, AR 72205, USA; 4 Pharmacology and Toxicology, University of Arkansas for Medical Sciences, 4301 West Markham St, Little Rock, AR 72205, USA; 5 Central Arkansas Veterans Healthcare System, John L McClellan Memorial Veterans Hospital, 4300 West 7th St., Little Rock, AR 72205, USA

## Abstract

**Background:**

Adeno-associated virus (AAV) type 2 is an important virus due to its use as a safe and effective human gene therapy vector and its negative association with certain malignancies. AAV, a dependo-parvovirus, autonomously replicates in stratified squamous epithelium. Such tissue occurs in the nasopharynx and anogenitals, from which AAV has been clinically isolated. Related autonomous parvoviruses also demonstrate cell tropism and preferentially replicate in oncogenically transformed cells. Combining these two attributes of parvovirus tropism, squamous and malignant, we assayed if AAV might replicate in squamous cervical carcinoma cell isolates.

**Results:**

Three primary isolates (PT1-3) and two established cervical cancer cell lines were compared to normal keratinocytes (NK) for their ability to replicate AAV. One isolate, PT3, allowed for high levels of AAV DNA replication and virion production compared to others. In research by others, four cellular components are known required for *in vitro *AAV DNA replication: replication protein A (RPA), replication factor C (RFC), proliferating cell nuclear antigen (PCNA), and DNA polymerase delta (POLD1). Thus, we examined PT3 cells for expression of these components by DNA microarray and real-time quantitative PCR. All four components were over-expressed in PT3 over two representative low-permissive cell isolates (NK and PT1). However, this super-permissiveness did not result in PT3 cell death by AAV infection.

**Conclusion:**

These data, for the first time, provide evidence that these four cellular components are likely important for AAV *in vivo *DNA replication as well as *in vitro*. These data also suggest that PT3 will be a useful reagent for investigating the AAV-permissive transcriptome and AAV anti-cancer effect.

## Background

As adeno-associated virus (AAV) increases in popularity as a gene therapy vector [1-6] we need to improve our understanding of the molecular biology of AAV replication. This will allow for better manipulation of AAV replication and, ultimately, should greatly boost rAAV production. Furthermore, while certain groups fail to see a correlation [7-9], the vast majority of epidemiologic, animal, and tissue culture studies strongly suggest that AAV inhibits the carcinogenesis process [10-29]. Moreover, there is a long history of AAV functioning as an autonomous parvovirus during specific circumstances. Yakobson et al. (1987) first observed the ability of AAV to replicate productively without helper virus in cells at low levels [30]. Others have demonstrated that a few cell lines, such as COS-7 cells, would allow for autonomous AAV replication [30-32]. All of these early studies utilized oncogenically transformed cells and in most circumstances the cells had to be treated with a genotoxic/synchronizing agent to achieve low level AAV replication. In a more recent study Wang and Srivastava (1998) demonstrated that mutation of the Rep78 binding site within the AAV p5 promoter allowed for low levels of autonomous AAV replication without genotoxic agents in HeLa cells [33].

We have been studying autonomous AAV replication in differentiating primary normal keratinocytes (NK) as they form a stratified squamous epithelium (SSE) [34-36]. AAV virus particle arrays have been identified in the nucleus of AAV infected differentiated keratinocytes with no concurrent adenovirus infection [34]. We hypothesized that AAV might replicate autonomously in SSE as AAV has been isolated from SSE at multiple body sites, including the anogenital region and the nasopharynx [37-39]. In continuing these studies primary squamous cervical cancer isolates and cell lines were surveyed for their ability to allow for AAV DNA replication. One primary isolate, PT3, was identified which allowed for 10 fold higher AAV DNA replication levels than NK and other cervical cancer cell lines [40]. In this study no genotoxic or cell synchronizing agents were used. The PT3 AAV super-permissive cell isolate offers us a unique reagent which might be useful in several ways. One use is to identify cellular genes that are needed for AAV autonomous replication by comparing the PT3 transcriptome to cells which allow only low AAV replication levels. The discovery of cellular helper and inhibitor genes for AAV will likely be very useful for increasing production of recombinant AAV virus for human gene therapy as well as for a better understanding of AAV's life cycle.

An important part of the molecular biology of AAV are the cellular proteins intimately involved in AAV DNA replication. In fact, a series of such proteins have already been identified to be directly involved in AAV *in vitro *DNA replication. These cellular components are replication protein A (RFA), replication factor C (RFC), proliferating cell nuclear antigen (PCNA), and DNA polymerase delta (PolD1)[41,42]. These proteins have also been shown to help minute virus of mice (MVM), an autonomous parvovirus [43]. To our knowledge only PT3 has been described as being super-permissive for AAV replication. Thus, to better characterize PT3, in this study we analyzed the RNA expression of these known replication proteins in PT3 cells compared to normal keratinocytes (NK) and another primary cervical cancer isolate, PT1. These latter two cell types allow only much lower levels of AAV DNA replication. It was found that all 4 of these cellular replication components are up-regulated in high AAV-permissive PT3 versus low-permissive PT1 or NK.

## Results

### AAV2 replicates significantly higher in PT3 cells

**Figure 1:**
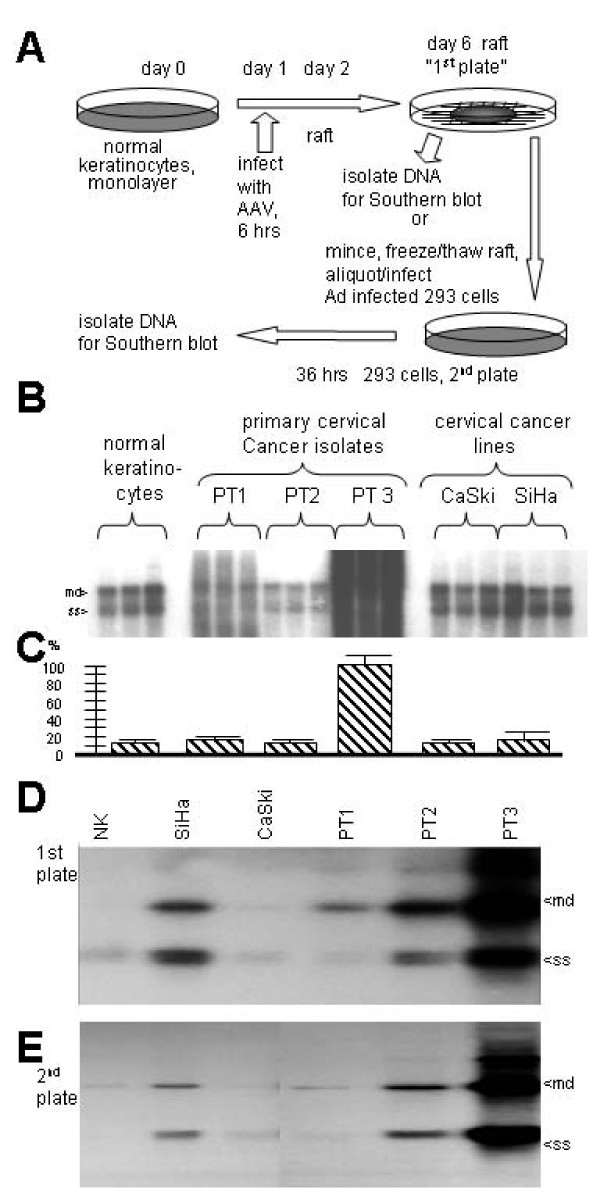
**High AAV replication and virion production in PT3 cells**. Equal numbers of the indicated cells were infected with AAV, cultured in the organotypic epithelial raft system and analyzed for AAV DNA replication and virion production as described in the materials and methods section. **A **shows the experimental scheme. **B **shows the resulting level of AAV DNA replication in a "first plate" experiment done in triplcate. Note that PT3 allows for the highest level of AAV DNA replication. **C **shows a densitometric quantification of the experiment shown in B. **D **shows the resulting level of AAV DNA replication in a "first plate" experiment, similar to that done in B, however the monomer duplex (md) and single stranded (ss) bands are not as overexposed as in B. **E **shows the level of AAV virion production by infection and replication in a "second plate" of adenovirus-infected 293 cells. Again, note that PT3 allows for the highest level of AAV virion production.

AAV has been isolated from SSE of the anogenitals and autonomous parvoviruses preferentially replicate in malignant cells. Thus, to test the hypothesis that AAV preferentially replicates in cervical cancer cells we compared three primary cervical cancer isolates and two archival cervical cancer cell lines to normal primary human foreskin keratinocytes (NK) for the ability to allow AAV autonomous replication and virion production within the organotypic epithelial raft culture system. All of these cells, except the normal keratinocytes, contain human papillomavirus type 16 (HPV-16) DNA. The NK cells represent a mixed culture of cells isolated from multiple individuals. The six types of cells were infected with AAV, transferred into the raft culture system to form a stratified squamous epithelium, harvested on day 6, DNA extracted, and analyzed by Southern blot. Two types of analyses were done as depicted in Figure 1A. First, AAV DNA replication was analyzed in the various squamous cell lines as SSE rafts, as a "first plate" analysis. Second, AAV virion production was measured by generating putative AAV virus stocks from equivalent "first plate" rafts and then a portion was used to infect a "second plate" of adenovirus-infected HEK293 cells. Any AAV DNA replication in the 293 cells would be due to AAV virions produced in the first plate rafts.

The Southern blot analysis of AAV replication directly in the first plate rafts is shown in Figure 1B. As can be seen, of the six cell types one isolate showed an unusually high level of AAV replication compared to other isolates. PT3 allowed for approximately a 10 fold higher level of AAV DNA replication compared to all other cervical cancer cell lines by densitometric analysis. All the other cervical cancer lines, and normal keratinocytes, also demonstrated AAV replication, but at a much low level. A quantification of the DNA replication levels is shown in Figure 1C. These results are comparable to a similar first plate raft experiment of AAV DNA replication shown in Figure 1D. However, coupled with this experiment is a second plate analysis of AAV virion production as shown Figure 1E. Note that PT3 was, in addition to higher AAV DNA levels, also demonstrated higher levels of virion production as well. Thus, PT3 is super permissive for complete AAV's full life cycle.

### Gene expression analysis with normalization to ACTB, GAPDH, or HG-U133A housekeeping genes

As PT3 allowed much higher levels of AAV replication we expected these cells to over express cellular components PCNA, POLD1, RFC, RPA1, and RPA [41,42]. Thus the transcriptome of PT3, representing the high AAV replication scenerio, was compared to low/normal AAV replication cell types PT1 and NK by DNA microarray analysis. Total RNA prepared from PT3, PT1 and NK was examined for the expression levels of Affymetrix HG-U133A (14,500 human genes). The RNA samples were isolated in-house and sent to the University of Iowa DNA Core for analysis. Three different methods for data normalization using ACTB, GAPDH, and Affymetrix U-133A housekeeping expression, respectively were utilized.

In data normalization methods using ACTB as a control housekeeping gene, all genes (6104 probe sets) we identified 1781 probe sets that changed at least 2-fold between PT3 and non-PT3. We also found 1311 up-regulated probe sets in PT3 and 470 down-regulated probe sets that changed at least 2-fold in either PT1 or NK. A total of 1781 probe sets pointed at differently expressed genes. Seven genes, members of four critical cellular components identified as essential for AAV DNA replication [41,42], were up-regulated in PT3 compared to PT1 and NK cells. These genes included PCNA, POLD1, RFC3, RFC4, RFC5, RPA1, and RPA2 (Table 1), when normalized to ACTB.

**Table 1:** Expression analysis of PCNA, POLD1, RFC and RPA using three different housekeeping controls.

Probe set	Description	Gene symbol	PT3			Non-PT3			Fold Differences		
			
			ACTB	GAPDH	U133-A	ACTB	GAPDH	U133-A	ACTB	GAPDH	U133-A
201202_at	proliferating cell nuclear antigen	PCNA	13.4	13.5	13.7	11.7	11.8	12.3	3.2	3.2	2.6
203422_at	polymerase (DNA directed), delta 1	POLD1	11.1	11.2	11.3	9.9	10.0	10.2	2.2	2.3	2.2
204128_s_at	replication factor C (activator 1) 3, 38 kDa	RFC3	11.4	11.5	11.6	9.4	9.4	9.9	4.0	4.0	3.2
204127_at	replication factor C (activator 1) 3, 38 kDa	RFC3	12.3	12.3	12.5	10.7	10.7	11.2	3.0	3.0	2.5
204023_at	replication factor C (activator 1) 4, 37 kDa	RFC4	13.3	13.4	13.6	11.3	11.4	11.9	4.0	4.0	3.3
203209_at	replication factor C (activator 1) 5, 36.5 kDa	RFC5	11.4	11.4	11.6	10.0	10.1	10.5	2.6	2.6	2.1
201528_at	replication protein A1, 70 kDa	RPA1	11.9	12.0	-	10.8	10.9	-	2.1	2.1	-
201529_s_at	replication protein A1, 70 kDa	RPA1	12.3	12.4	-	11.2	11.3	-	2.0	2.0	-
201756_at	replication protein A2, 32 kDa	RPA2	12.5	12.6	12.7	10.9	11.0	11.5	2.9	2.9	2.3

Three difference methods for data normalization using ACTB, GAPDH, and Affymetrix U-133A housekeeping genes, respectively, were utilized.

Normalization of all probe sets (5789 probe sets) to expression of GAPDH as a control gene revealed 1440 probe sets that were up-regulated, and 429 probe sets that were down-regulated, in PT3 compared to PT1 and NK cell lines, for a total of 1869 genes of all differently expressed genes. Yet again the same seven AAV-critical genes were up-regulated in PT3 compared to PT1 and NK, (Table 1), this time when normalized to GAPDH. These data provide evidence that the cellular components reported to be involved in AAV *in vitro *DNA replication may also be involved *in vivo *AAV DNA replication as well. Furthermore these data suggest a mechanistic explanation as to why PT3 allows high AAV DNA replication.

Affymetrix U-133A housekeeping genes normalization, across all probe sets (4581 probe sets) on the array, revealed 791 up-regulated and 687 down-regulated transcripts in PT3 compared to PT1 and NK cell lines, for a total of 1478 probe sets of all differently expressed genes. Again six of seven of the same AAV-critical genes were up-regulated in PT3 compared to PT1 and NK, (Table 1), this time when normalized to a broad series of housekeeping genes. Using this third control analysis, RPA1 dropped out due to lack of statistical significance. Similar analyses were made for cellular helicases and DNA polymerase α, which have been suggested to be involved in AAV DNA replication. As can be seen the data suggests that cellular helicases DHX9 and RECQL were up-regulated in PT3 compared to PT1 and NK, however DNA2L was down-regulated (Table 2). The data also suggests that cellular DNA polymerase α was up-regulated in PT3 compared to PT1 and NK (Table 3).

**Table 2:** DNA helicase expression.

Probe set	Description	Gene symbol	PT3			Non-PT3			Fold Differences		
			
			ACTB	GAPDH	U133-A	ACTB	GAPDH	U133-A	ACTB	GAPDH	U133-A
34063_at	RecQ protein-like 5	RECQL5	-	-	-	-	-	-	-	-	-
202420_s_at	DEAH (Asp-Glu-Ala-His) box polypeptide 9	DHX9	-	-	-	-	-	-	-	-	-
205091_x_at	RecQ protein-like (DNA helicase Q1-like)	RECQL	-	-	-	-	-	-	-	-	-
210309_at	RecQ protein-like 5	RECQL5	-	-	-	-	-	-	-	-	-
210568_s_at	RecQ protein-like (DNA helicase Q1-like)	RECQL	-	-	-	-	-	-	-	-	-
211468_s_at	RecQ protein-like 5	RECQL5	-	-	-	-	-	-	-	-	-
212105_s_at	DEAH (Asp-Glu-Ala-His) box polypeptide 9	DHX9	10.3	10.4	-	9.1	9.1		2.3	2.4	
212107_s_at	DEAH (Asp-Glu-Ala-His) box polypeptide 9	DHX9	-	-	-	-	-	-	-	-	-
212917_x_at	RecQ protein-like (DNA helicase Q1-like)	RECQL	10.6	10.7	-	9.5	9.6		2.2	2.3	-
212918_at	RecQ protein-like (DNA helicase Q1-like)	RECQL	-	-	-	-	-	-	-	-	-
213520_at	RecQ protein-like 4	RECQL4	-	-	-	-	-	-	-	-	-
213647_at	DNA2 DNA replication helicase 2-like (yeast)	DNA2L	8.6	8.7	8.7	10.2	10.2	10.2	-3.0	-2.8	-2.8
213878_at	similar to CG10721-PA	LOC642732	-	-	-	-	-	-	-	-	-
221686_s_at	RecQ protein-like 5	RECQL5	-	-	-	-	-	-	-	-	-

Three different methods for data normalization using ACTB, GAPDH, and Affymetrix U-133A housekeeping genes, respectively were utilized.

**Table 3:** Expression of DNA polymerase alpha.

Probe set	Description	Gene symbol	PT3			Non-PT3			Fold Differences		
			
			ACTB	GAPDH	U133-A	ACTB	GAPDH	U133-A	ACTB	GAPDH	U133-A
204441_s_at	Polymerase (DNA directed), alpha 1	POLA1	-	-	-	-	-	-	-	-	-
204835_at	Polymerase (DNA directed), alpha 1	POLA1	11.7	11.8	11.8	10.1	10.1	10.1	2.9	3.1	3.1

Three different methods for data normalization using ACTB, GAPDH, and Affymetrix U-133A housekeeping genes, respectively were utilized.

### Comparison of Normalization Techniques

Based on the number of transcripts identified as differentially expressed, the three techniques used to normalize the array data could be ordered by the number of genes identified as differentially expressed as follows: GAPDH (1869 probe sets) > ACTB (1781 probe sets) > U-133A (1478 probe sets). Although the three array normalization methodologies differed in the number of genes defined as down- or up-regulated in expression in PT3 compared to PT1 and NK cell lines, all identified the same 7 up-regulated genes (PCNA, POLD1, RFC3, RFC4, RFC5, RPA1, and RPA2) except RPA1 in normalization using HG-U133A housekeeping genes (Table 1). This finding suggested that these seven genes were clearly differentially over-expressed in PT3 versus PT1 and NK cell lines.

### Verification of microarray results by real-time quantitative PCR

**Figure 2:**
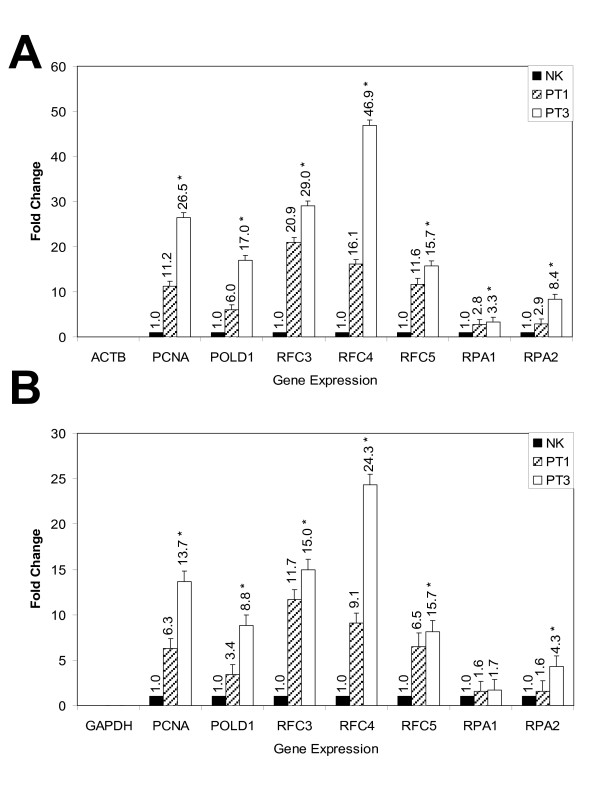
**Real-time quantitative PCR analysis of differentially expressed transcripts in NK, PT1 (upward diagonal bars) and PT3 (open bars)**. Data are expressed relative to ACTB (2A) and GAPDH (2B) mRNA and (*) presented *p *< 0.05. Fold-expression changes were calculated using the equation 2^-ΔΔCT ^[5]. Error bars for each column in the plot provided that the associated expression level was calculated from 3 replicates. The error bars display the calculated maximum (RQMax) and minimum (RQMin) expression levels that represent standard error of the mean expression level (RQ value). Collectively, the upper and lower limits defined the region of expression within which the true expression level value was likely to occur. The error bars was based on the RQMin/Max confidence level. The number associated with each bar indicates the linear fold-change of mRNA expression in PT1 and PT3 relative to NK for comparison.

**Figure 3:**
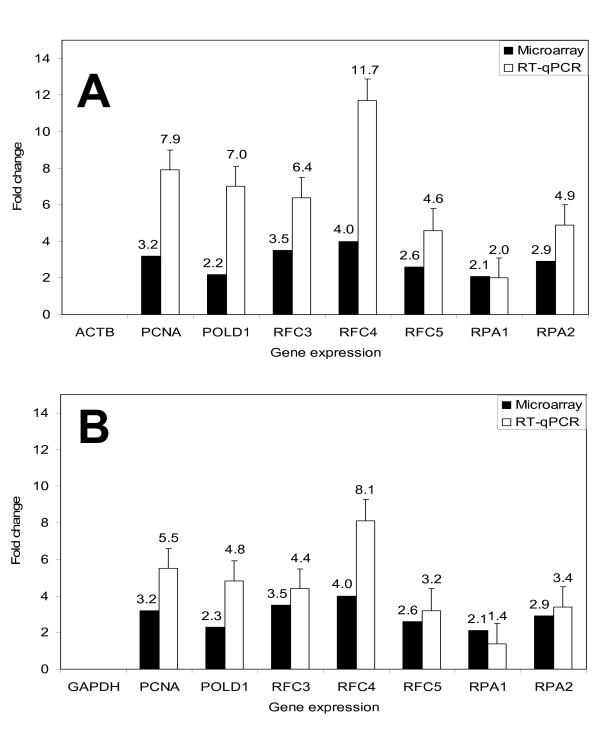
**Real-time quantitative PCR analysis (open bars) of genes selected from the microarray (closed bars) in PT3 and Non-PT3**. Data are expressed relative to ACTB (3A) and GAPDH (3B) mRNA and (*) presented *p *< 0.05. The gene expression levels were sorted by detector. Gene expression levels for PT3 are indicated by the black bar. This color also indicates the sample in the RQ sample grid and the RQ results panel plots. Because NK samples are used as calibrator, the expression levels are set to 1. But because the gene expression levels were plotted as log_10 _values (and the log_10 _of 1 is 0), the expression level of the calibrator samples appear as 0 in the graph. In addition, because the relative quantities as the targets are normalized against the relative quantities of the reference genes, the expression level of the reference genes is 0, that is, there are no bars for ACTB and GAPDH. Fold-expression changes were calculated using the equation 2^-ΔΔCT ^[5]. Error bars for each column in the plot provided that the associated expression level was calculated from 3 replicates. The error bars display the calculated maximum (RQMax) expression levels that represent standard error of the mean expression level (RQ value). Collectively, the upper and lower limits defined the region of expression within which the true expression level value was likely to occur. The error bars was based on the RQMin/Max confidence level. The number associated with each bar indicates the linear fold-change of mRNA expression in PT3 relative to Non-PT3 for comparison.

As we did the microarray analysis using a single mRNA isolation/cDNA probe analysis, we needed to verify the transcriptional over-expression of these seven genes by real-time quantitative PCR. To determine the optimum amount of cDNA template in initial experiments, we performed undiluted, 1:10 diluted, and 1:100 diluted cDNA template in parallel. Gene expression of NK, PT1 and PT3 cDNA templates were normalized to the Ct value of reference genes: GAPDH or ACTB, respectively, to calculate the Ct values of PCNA, POLD1, RFC3, RFC4, RFC5, RPA1, and RPA2. We used NK as calibrator (Figure 2A and 2B). The RT-qPCR results confirmed the microarray results, that PCNA, POLD1, RFC3, RFC4, RFC5, RPA1, and RPA2 were over-expressed in PT3 (at least a 1.8 fold difference between two groups [PT3 vs Non-PT3]). The relative quantitative expression of the 7 genes between PT3 and Non-PT3 samples was set at a significance level of 0.05. To see the comparative gene expression levels of PCNA, POLD1, RFC3, RFC4, RFC5, RPA1, and RPA2, comparing the microarray and qPCR results, we used non-PT3 (NK and PT1) cells as the calibrator (Figure 3A and 3B).

### Super-permissiveness does not correlate with cytotoxicity

**Figure 4:**
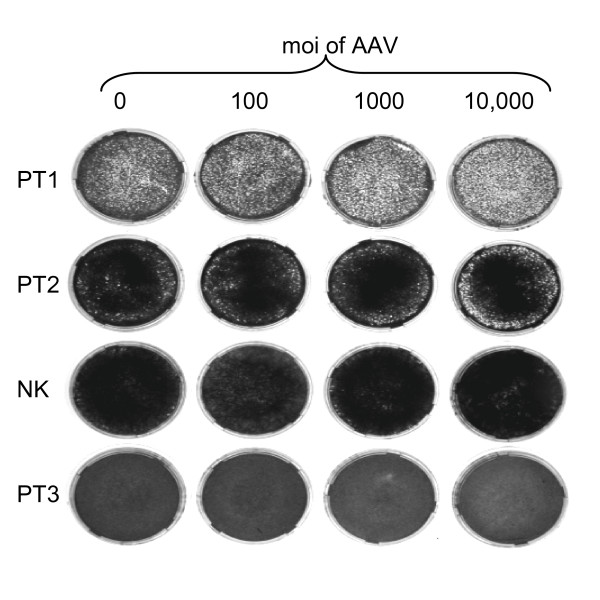
**Lack of cytotoxicity by AAV**. Various squamous cell isolates were grown in culture and infected with AAV2 virus as indicated. Note that increasing mois of AAV2 did not result in increased cell toxicity of PT3, and had only minimal effects on the cell growth of the other cells. Shown is a representative experiment of three done.

It has been reported in CNN in 2005, in work done by Craig Meyers, that AAV preferentially kills cancer cells http://www.cnn.com/2005/HEALTH/06/22/cancer.virus/. This reported cancer cell killing may be related to parvovirus replication as certain parvoviruses have been reported to preferentially replicate in malignant cells [44]. Thus we tested the high and low AAV-permissive cells for their sensitivity to killing by AAV infection. The results are shown in Figure 4 and demonstrate that PT3 was not preferentially sensitive to killing by AAV2 infection compared to other squamous cells.

## Discussion

Earlier studies by Ni *et al *and Nash *et al *identified a number of cellular components which are required for *in vitro *AAV DNA replication using both adenovirus-infected and uninfected cell extracts [41,42]. These cellular components, found to be critical, include PCNA, RFC, RPA and DNA polymerase delta (POLD1). This study demonstrates that the PT3 primary cervical cancer cell isolate, which is super-permissive for AAV replication [40], over-expresses all four of these components, when compared with PT1/NK. Thus, the data presented here are fully consistent with the earlier *in vitro *studies, but now extend these studies into the context of the living cell. These data also further characterize the primary cervical cancer isolate PT3 and confirms the ability of AAV to replicate in SSE, now including malignant cells [34-36]. It is also confirmed that AAV2 variably replicates in multiple cervical cancer isolates [40]. Thus far, to our knowledge, only the PT3 isolate has been described as super-permissive for AAV replication, this being when compared to a variety of cells of squamous origin.

Both the Affymetrix DNA microarray data and real-time quantitative PCR results demonstrated that all four of these cellular components were over expressed in PT3 cells. POLD1 and PCNA were strongly over-expressed in PT3. Moreover, multiple RFC and RPA family members were over-expressed in PT3. Thus, these data support the unusual phenotype of PT3 cells and suggest their use as a unique reagent for identifying critical genes involved in AAV replication. This phenotype also suggests the possibility that PT3, itself, may be useful as a platform for rAAV production. One issue against this idea is that AAV replication in PT3 takes place during cellular differentiation (induced by air interface and calcium). During such differentiation cells of keratinocyte origin become highly keratinized and become physically very stable, somewhat similar to a ''piece of plastic''. This is problematic for the efficient isolation of rAAV from keratinized PT3 cells. However this possibility is worth investigating.

Ni *et al *and Nash *et al *[41,42] identified POLD1 as the central DNA polymerase, which is a leading strand DNA polymerase, the main mechanism through which AAV DNA replication takes place. The need of PCNA and RFC is also compatible with POLD1 as the main AAV-polymerase as PCNA is the processivity factor for POLD1, and RFC is known to assemble PCNA onto 3'OH primers. RPA was not found essential when using adenovirus-infected cell extracts, in contrast to uninfected cell extracts [41]. In any case these data are also consistent with Christensen and Tattersall [43] who found that these same four proteins (POLD1, RPA, PCNA, and RFC) were the minimum cellular factors required for MVM DNA rolling-circle replication when using a 3'-dimer junction. However their *in vitro *reactions also included MVM NS1 protein and cellular PIF protein.

In the latest study by Nash *et al *[41] it was mentioned that there is one additional protein component (present in P-Cell IA) which was needed but was unidentified. It was further speculated that it was a cellular helicase. To approach this question we revisited the PT3vsPT1/NK DNA microarray data to observe if particular DNA helicases or overall helicase activity was higher in PT3. This approach seems valid as even though we have not done the usual triple-DNA microarray analysis, the real-time quantitative PCR expression data fully confirmed the DNA microarray results across multiple genes. Thus, the Affymetrix microarray data we have in hand appears worthy of study for gleaning suggestive information on the AAV-permissive transcriptome. It was found, as shown in Table 2, that the overall helicase activity was not significantly different in PT3 cells, with two helicases being up-regulated and one down-regulated in PT3 versus NK/PT1.

While POLD1 was clearly found required for AAV *in vitro *replication by Nash et al [41] there is a possibility the DNA Polymerase alpha might be involved in certain "alternative" forms of AAV DNA replication, such as through the use of internal origins of replication [45]. Both SV40 and parvovirus H-1 are able to use Polymerase alpha for replication [46,47]. To approach this question we revisited the PT3vsPT1/NK DNA microarray data to observe if DNA polymerase alpha was higher in PT3. The results of the Affymetrix data are shown in Table 3, and suggest that DNA polymerase alpha is also significantly up-regulated in PT3 over PT1 and NK. However, the importance of this up-regulation, if any, is not yet determined.

One question which arises from this data is how or if the four components are coordinately up-regulated in PT3 cells. As we are analyzing the RNA expression of these genes transcriptional regulation is the most likely explanation and it is possible that a common transcription factor regulates them all. Possible examples for such coordinated transcriptional regulation include transcription factors hDREF, CFDD, p53, and Sp1, among others. One anecdotal finding needs to be mentioned about the PT3 cell isolate, that being its high sensitivity to culture conditions. PT3 cell cultures, when grown side by side with PT1 and NK, would go into a period of en masse cell death if not fed in a timely fashion, or kept out of the incubator for too long. PT1 and NK cells were resistant to such die-offs under similar culture conditions. In any case, the isolation and characterization of the novel PT3 cell line gives us a unique reagent to investigate the optimal cellular transcriptome needed for AAV2 replication. Such knowledge will be useful for understanding AAV molecular biology, for generating high yield rAAV virus for gene therapy, and for understanding AAV's anti-cancer properties.

## Conclusion

The novel cell line PT3 is super-permissive for AAV DNA replication and over-expresses DNA polymerase δ, PCNA, RFC and RPA. This is important as *in vitro *studies by Ni *et al *and Nash *et al *have identified these same cellular components as being involved in AAV DNA replication *in vitro*. Our *in vivo *data and the *in vitro *data of others, together, strongly suggest that the PT3 cell line is a unique reagent which can be used to investigate the optimal cellular transcriptome which is needed for AAV replication. The further "mining" of PT3vsPT1/NK microarray data to intimate additional AAV-relevant genes will ultimately give us better understanding of AAV molecular biology, better understanding of AAV's anti-cancer properties, and ultimately allow for higher yields in the production of rAAV virus for gene therapy.

## Methods

### Cell lines

Primary human foreskin keratinocytes (NK) were purchased from Clonetics Inc.(San Diego, CA). PT1, PT2, and PT3 primary cell lines were isolated from three cervical cancer patients as described previously [48]. These cervical cancer isolates were at approximately passage 10–15 when used in these experiments. CaSki and SiHa cervical cancer cell lines were purchased from American Type Culture Collection (Rockville, MD). All the cells were cultured in keratinocyte serum-free medium (Invitrogene, Carlsbad, CA) in 37°C under 5% CO_2 _prior to raft formation.

### AAV replication in squamous cells using the organotypic epithelial raft cultures

On day 1, 10^6 ^normal primary keratinocytes, three primary cervical cancer cells and CaSki, SiHa cells were infected with 10^8 ^infectious units of wild type AAV-2 virus (multiplicity of infection [moi] = 100). On day 2 the cells were trypsinized, plated onto J2-containing collagen rafts as described previously [34-37]. On day 3 these organotypic skin rafts were raised to the air interface and allowed to form an SSE over a period of 3 days (day 6 overall) using E medium. Southern blot analysis was done to detect AAV DNA replication. Rafts were harvested on day 6. After washing with phosphate-buffered saline (PBS, 137 mM NaCl, 10 mM Phosphate, 2.7 mM KCl, pH 7.4.) twice, all the rafts were minced and lysed. Total DNA was extracted and 10 μg of total cellular DNA were analyzed for AAV DNA replication levels by agarose gel electrophoresis, Southern blotting, and probing with ^32^P-AAV Cap DNA probe to pick up only the wt AAV genome. Finally, a quantification of the Southern blot was done by densitometric analysis using an Alpha Imager 2000 (Alpha Innotech Corporation, San Leandro, CA). The densitometric data was quantified using AlphaImager™ 2000 software. Densitometric data was analyzed by the unpaired *t-test *and presented as mean ± standard error (SE).

### "Second plate" analysis of AAV virion production

Instead of harvesting the keratinocyte rafts for the analysis of AAV DNA replication on day 6, in certain experiments the SSE rafts were analyzed for AAV virion production by the infection of a "second plate" of adenovirus infected HEK293 cells. Putative AAV virus stocks were generated by freezing day 6 rafts and grinding the rafts with mortar and pestle. The remains of the raft were placed in one ml of DMEM medium, vortexed for 1 minute and centrifuged at 8,000 g for 15 minutes to remove debris, and the supernatant was filtered through a 20 um filter. One third of the putative virus stock was used to infect a 6 cm plate on 80% confluent monolayer HEK293 cells. These cells were also infected with Ad helper virus at an moi of 5. Any AAV infectious units produced in the original raft would be amplified in the Ad-infected 293 cells. After 36 hours of infection total DNA was extracted and 10 μg of total cellular DNA were analyzed for AAV DNA replication levels by agarose gel electrophoresis, Southern blotting, and probing with ^32^P-AAV cap DNA probe.

### AAV2 cytotoxicity in cervical cancer cell isolates

AAV2 virus stock was serially diluted with Dulbecco's medium (supplemented with 10% FBS and 100 U/ml penicillin). Normal keratinocytes and three primary cancer cell lines (PT1, PT2 and PT3) were seeded (4 × 10^5^/dish) one day prior to infection with serially diluted wild type AAV 2 in 1 ml culture media at a multiplicity of infection (moi) of 100, 1,000, 10,000 AAV particles. Culture media was replaced with E medium after overnight incubation at 37°C and were incubated for additional 6 days with fresh media at one day interval. At day 7 the cells were washed with PBS, fixed in formaldehyde and stained with methylene blue. The experiment was done three times.

### Total RNA extraction and cDNA synthesis

For real-time quantitative PCR (qPCR), total RNA samples from 1 × 10^6 ^cultured cells was extracted from NK, PT1 and PT3 cell lines using Total RNA Purification System Kit (Invitrogen, USA) according to the manufacturer's protocol. Concentration of mRNA was quantified using NanoDrop^® ^ND-1000 Spectrophotometer (NanoDrop technology, USA). One μg was transcribed into first strand cDNA using Transcriptor First Strand cDNA Synthesis Kit (Roche, Indianapolis, IN) according to the manufacturer's protocol. For Affymetrix microarray analysis, total RNA was isolated from NK, PT1 and PT3 cell lines using Trizol (Invitrogen, Carlsbad, CA) according to the manufacturer's protocol. After treatment with 5 U/μg of RNase-free DNase I at 37°C for 1 hour, all the samples were frozen in and sent to University of Iowa DNA facility for microarray analysis. After cDNA synthesis, samples were applied to a Human Genome GeneChip HG-U133A (Affymetrix Inc. Santa Clara, CA).

### Array filtering and significant expressed gene identification

Microarray data in the form of CEL files were imported into BRB ArrayTools developed by Dr. Richard Simon and Amy Peng Lam http://linus.nci.nih.gov/BRB-ArrayTools.html. HG-U133A microarray raw expression intensities of NK, PT1, and PT3 data were scaled to a target intensity of 100 units, normalized independently, using the robust multichip average (RMA) algorithm for the quantification of the expression level of target genes, and passed by the filtering and subletting criteria with any one absent (A) or marginal call (M). Genes that had more than 50% missing data across all observations were excluded from the analysis. Also, we selected those genes with an expression level of ≥ 20 in ≥ 25% of samples. Fold change has been transformed based on log_2 _(PT1/NK), log_2_(PT3/NK), log_2 _(PT3/PT1), log_2 _(PT3/non-PT3), respectively. Fold change above 2.0 was defined as differentially expressed genes between two cell lines, where it is meet fold >2 SD (above 97% confidence).

### Real-time quantitative PCR

Validation of differential expressed genes was done by real-time quantitative PCR (RT-qPCR). RT-qPCR assays were performed using the Applied Biosystems 7500 Systems (Applied Biosystems, USA). Each sample was run in triplicate to ensure quantitative accuracy. We used Human Universal ProbeLibrary from Roche Applied Science. Assay specificity was attained through the combination of specific primers designed from ProbeFinder https://www.roche-applied-science.com) web-based software. Seven genes, plus two reference genes, with their specific primers, and PCR product size information for real-time quantitative PCR validation are listed in Table 4.

**Table 4:** Primer information for real-time qPCR.

Gene name	Size (bp)	Forward primer sequence (5' – 3')	Reverse primer sequence (5' – 3')
***PT3-inducible genes***			
*PCNA*	66	GAACTGGTTCATTCATCTCTATGG	TGTCACAGACAAGTAATGTCGATAAA
*POLD1*	107	AGGTAGTACTGCGTGTCAATGG	CCCTACGTGATCATCAGTGC
*RFC3*	69	CACAGTAGATAACACGTGGCAAA	AGTAGGTGCTTGGCGGTTC
*RFC4*	77	TTCCAGGTGGTCCGTAAAAC	AGAAGTGGTTGCAGTGCTGA
*RFC5*	84	TGTGGCCGGTATTTTTCAAC	GGAGACCTCAGCACTCAAGC
*RPA1*	65	CCGTAGTAATGGGACGGATG	GCAGAAGGGGGATACAAACA
*RPA2*	95	TTCCAGAATATGTGTGGTGAACTC	GCCACCTGAGATCTTTTCAGA
***Reference genes***			
*ACTB*	*97*	CCAGAGGCGTACAGGGATAG	CCAACCGCGAGAAGATGA
*GAPDH*	*66*	GCCCAATACGACCAAATCC	AGCCACATCGCTCAGACAC

All PCR reactions were carried out in a final volume of 50 μl containing 1× of FastStart Universal SYBR Green Master [ROX] (Roche Applied Science, USA) with a reporter dye at the 5' end (FAM) and a quencher at the 3' end, 300 nM of each gene specific primers (Table 1), 50 ng cDNA, and then added sterile deionized water. The standard cycling condition was 50°C for 2 min, 90°C for 10 min, followed by 40 cycles of 95°C for 15 s and 60°C for 1 min.

To quantify the relative expression of each gene, real-time qPCR data were first reported as (1) NK: PT1 and PT3 as well as (2) non-PT3 (NK and PT1):PT3 ratios. The comparative threshold cycle (C_t_) values for NK, PT1, and PT3 samples were normalized for reference genes (ΔC_t _= C_t target _- C_t ACTB or GAPDH_) and compared with a calibrator using the ΔΔCt method [49]. As calibrator, the average Ct value of each gene in all samples grouped together was taken. All reported real-time quantitative PCR reactions were performed and analyzed using an ABI 7500 System SDS Software Ver1.3 (Applied Biosystems, USA). Fold units were calculated dividing the expression fold changes of the candidate genes by the expression fold changes of the reference gene (ACTB or GAPDH).

### Statistical analysis

Comparison of the relative quantitative expression of the 7 genes between PT3 and Non-PT3 samples was done with an unpaired *t-test *comparing two groups, with a significance level of 0.05 using Microsoft Excel 2003 program and presented as mean ± standard error (SE). All real time quantitative PCR were performed in triplicate to ensure quantitative accuracy.

## List of abbreviations

AAV: Adeno-associated virus; ACTB: beta-actin; DNA: deoxyribonucleic acid; POLD1: DNA polymerase delta; MVM: minute virus of mice; GAPDH: glyceraldehyde-3-phosphate dehydrogenase; NK: normal keratinocytes; PCNA: proliferating cell nuclear antigen; RT-qPCR: real time quantitative polymerase chain reaction; RFC: replication factor C; RFA: replication protein A, mRNA: messenger ribonucleic acid; NaCl: sodium chloride; SSE: stratified squamous epithelium

## Competing interests

The authors declare that they have no competing interests.

## Authors' contributions

BYK carried out the computational analysis of the microarray data and the real time PCR. HY, YL, and NA carried out the replication and microarray experiments, SB carried out the cytotoxicity assay. RBM, AGB, and PLH critically analyzed the results and assisted in writing the paper. PLH designed the study and was the primary writer of the manuscript. All authors have read and approved the final version of the manuscript.
